# The effect of a pre- and postoperative orthogeriatric service on cognitive function in patients with hip fracture: randomized controlled trial (Oslo Orthogeriatric Trial)

**DOI:** 10.1186/1741-7015-12-63

**Published:** 2014-04-15

**Authors:** Leiv Otto Watne, Anne Cathrine Torbergsen, Simon Conroy, Knut Engedal, Frede Frihagen, Geir Aasmund Hjorthaug, Vibeke Juliebo, Johan Raeder, Ingvild Saltvedt, Eva Skovlund, Torgeir Bruun Wyller

**Affiliations:** 1 Oslo Delirium Research Group, Department of Geriatric Medicine, Oslo University Hospital, Oslo, Norway; 2 University of Oslo, Institute of Clinical Medicine, Oslo, Norway; 3 Department of General Internal Medicine, Oslo University Hospital, Oslo, Norway; 4 Department of Cardiovascular Sciences, University of Leicester School of Medicine, Leicester, UK; 5 Norwegian Centre for Ageing and Health, Vestfold Mental Health Trust, Vestfold, Norway; 6 Department of Orthopaedic Surgery, Oslo University Hospital, Oslo, Norway; 7 Department of Cardiology, Oslo University Hospital, Oslo, Norway; 8 Department of Anesthesiology, Oslo University Hospital, Oslo, Norway; 9 Department of Geriatrics, St. Olav Hospital, University Hospital of Trondheim, Trondheim, Norway; 10 Department of Neuroscience, Norwegian University of Science and Technology (NTNU), Trondheim, Norway; 11 School of Pharmacy, University of Oslo, Oslo, Norway

**Keywords:** Hip fracture, Orthogeriatrics, Delirium, Cognitive decline

## Abstract

**Background:**

Delirium is a common complication in patients with hip fractures and is associated with an increased risk of subsequent dementia. The aim of this trial was to evaluate the effect of a pre- and postoperative orthogeriatric service on the prevention of delirium and longer-term cognitive decline.

**Methods:**

This was a single-center, prospective, randomized controlled trial in which patients with hip fracture were randomized to treatment in an acute geriatric ward or standard orthopedic ward. Inclusion and randomization took place in the Emergency Department at Oslo University hospital. The key intervention in the acute geriatric ward was Comprehensive Geriatric Assessment including daily interdisciplinary meetings. Primary outcome was cognitive function four months after surgery measured using a composite outcome incorporating the Clinical Dementia Rating Scale (CDR) and the 10 words learning and recalls tasks from the Consortium to Establish a Registry for Alzheimer’s Disease battery (CERAD). Secondary outcomes were pre- and postoperative delirium, delirium severity and duration, mortality and mobility (measured by the Short Physical Performance Battery (SPPB)). Patients were assessed four and twelve months after surgery by evaluators blind to allocation.

**Results:**

A total of 329 patients were included. There was no significant difference in cognitive function four months after surgery between patients treated in the acute geriatric and the orthopedic wards (mean 54.7 versus 52.9, 95% confidence interval for the difference -5.9 to 9.5; *P* = 0.65). There was also no significant difference in delirium rates (49% versus 53%, *P* = 0.51) or four month mortality (17% versus 15%, *P* = 0.50) between the intervention and the control group. In a pre-planned sub-group analysis, participants living in their own home at baseline who were randomized to orthogeriatric care had better mobility four months after surgery compared with patients randomized to the orthopedic ward, measured with SPPB (median 6 versus 4, 95% confidence interval for the median difference 0 to 2; *P* = 0.04).

**Conclusions:**

Pre- and postoperative orthogeriatric care given in an acute geriatric ward was not effective in reducing delirium or long-term cognitive impairment in patients with hip fracture. The intervention had, however, a positive effect on mobility in patients not admitted from nursing homes.

**Trial registration:**

ClinicalTrials.gov NCT01009268 Registered November 5, 2009

## Background

More than 30% of individuals 65-years-old or older experience at least one fall each year, and the prevalence increases with age [1]. Ten percent of falls result in serious injuries [2], with hip fracture as one of the most feared consequences. In the European Union it was estimated that 615,000 new hip fractures occurred in 2010, and the number of hip fractures is expected to increase in the years to come [3].

Patients with hip fracture are often frail, have multiple co-morbidities including cognitive impairment, and there is usually polypharmacy [4]. To address these patients' needs, different models of orthogeriatric co-management have been developed. Models range from a limited consultation or liaison service through to integrated orthogeriatric units [5]. Few of these models have been evaluated in randomized controlled trials (RCTs), and the heterogeneity of interventions, outcomes and populations makes it difficult to draw conclusions regarding the superiority of one particular model [5-7]. Geriatric intervention might be especially beneficial in the vulnerable period prior to surgery, but most studies are limited to postoperative orthogeriatric intervention [8].

A common complication of hip fracture is delirium, a syndrome of acute change in cognition and alertness, and altered, often psychotic, behavior [9]. About 40% to 50% of hip fracture patients are reported to develop delirium in the peri-operative period [10]. Delirium is particularly common in patients with pre-existing dementia [11], despite which patients with dementia are often excluded from studies [12]. Delirium in the peri-operative phase is associated with increased risk of death, institutionalization and subsequent dementia [13]. Multifactorial intervention can prevent delirium in hip fracture patients [14-16], but it is not yet established if preventing delirium can reduce long-term cognitive decline.

In 2008, we established an orthogeriatric service at our hospital, comprising pre- and postoperative care of hip fracture patients in the acute geriatric ward. We evaluated this model by a RCT in which hip fracture patients receiving usual care in the orthopedic ward comprised the control group. We hypothesized that the intervention could prevent delirium-associated long-term cognitive decline and, thus, chose cognitive function four months after surgery as the primary outcome.

## Methods

### Project context

In 2008, orthogeriatric care at Oslo University Hospital was reorganized and became a part of the acute geriatric ward. The new service had the capacity to serve approximately half of the patients admitted with hip fracture. The remaining patients were treated in the orthopedic ward. To evaluate the new model, we randomly allocated patients between the acute geriatric and the orthopedic wards. The first hip fracture patient was admitted to the acute geriatric ward in June 2008 and after a pilot period inclusion in the study started in September 2009. The recruitment ended in January 2012. The study protocol containing further information is published elsewhere [17].

### Study design

We carried out a randomized, controlled, single-blind trial comparing pre- and postoperative orthogeriatric care integrated in the acute geriatric ward to usual care in the orthopedic ward. Inclusion and randomization took place in the emergency department, overseen by the duty orthopedic surgeon. Allocation was by sealed, opaque, numbered envelopes. Randomization was based on computer-generated random numbers (blocks of variable and unknown size) and was carried out by a statistician (ES) not involved in the clinical service. Randomization was stratified according to whether or not the patients were admitted from nursing homes. Included patients were transferred directly from the emergency department to the allocated ward, and had their entire hospital stay in the same ward except for time in the operating theater and a few hours in the postoperative care unit. Operative and anesthetic procedures were the same in the two groups.

### Study participants

All patients admitted acutely to Oslo University Hospital with a hip fracture (a femoral neck fracture, a trochanteric or a sub-trochanteric fracture) were eligible for inclusion. Patients were excluded if the hip fracture was a part of a high energy trauma (defined as a fall from higher than one meter) or if they were moribund on admission.

### Intervention and control

Patients randomized to intervention were treated in the acute geriatric ward (Table 1). This was a 20 bed ward, mainly admitting patients suffering from acute medical disorders superimposed upon frailty, co-morbidities and polypharmacy. The only surgical patients treated in the ward were the hip fracture patients included in the trial. On average during the inclusion period, two to four beds were used for hip fracture patients. The acute geriatric ward was regularly full or over-crowded. To avoid randomization violation, the ward was instructed to admit included hip fracture patients even if the ward was full. Thus, some hip fracture patients had to be treated in the corridor until a room was available, usually within the first 24 hours.

**Table 1:** Organization of treatment in the acute geriatric ward and the orthopedic ward

**Description of ward**	**Acute geriatric ward**	**Orthopedic ward**
Department	Clinic of Internal Medicine, Department of Geriatrics	Department of Orthopedic Surgery
Number of beds	20	52
Average number of beds occupied	101%	90%^a^
Organization of ward	Hip fracture patients spread among other medical patients	Hip fracture patients spread among other surgical patients
Staff-order (number per bed)		
- nurses	1	1.18
- nursing assistants	0.28	0.06
- physiotherapists	0.08	0.07
- occupational therapists	0.07	0
- nutritionists	available on request	0
- social worker	available on request	0.02
Interdisciplinary meetings	Daily	No
Intervention after discharge	Patients offered control at orthopedic outpatient clinic four months after surgery	Patients offered control at orthopedic outpatient clinic four months after surgery

^a^For the orthopedic ward, only figures from 2011 were available.

A key element of the intervention was a Comprehensive Geriatric Assessment (CGA) as a basis for treatment planning. All team members (geriatrician, nurse, physiotherapist and occupational therapist) were expected to assess patients during their first day on the ward, and the team had daily meetings to co-ordinate treatment and to plan discharge. Clinical routines were developed based on a literature search, experience from earlier orthogeriatric models and the pilot phase prior to the start of randomization. Checklists were printed out and made immediately available for the treatment team for each patient. Details about the clinical routines have been published [17] and included medication reviews, early and intensive mobilization, optimizing pre- and postoperative nutrition and early discharge planning.

The control group was treated in the orthopedic ward, a 52 bed ward admitting a range of elective and non-elective orthopedic patients. The staff-patient ratio was similar to that of the acute geriatric ward (Table 1). There were, however, no multidisciplinary meetings and no geriatric assessments. Early mobilization was emphasized, and hip fracture patients were seen by a physiotherapist soon after surgery. The postoperative care unit was within the orthopedic ward, where all patients (including those allocated to intervention) were observed after surgery.

All patients included in the trial were offered a control in the orthopedic outpatient clinic four months after surgery. There was no additional intervention after discharge from hospital.

### Measurements

Social and demographic information was collected during the acute stay. Information regarding surgical and anesthetic procedures, medical diagnoses (Charlson comorbidity index [18]), drug use and complications was also collected. Proxies were interviewed regarding pre-fracture Activities of Daily Living (Barthel ADL Index (BADL [19]) and Nottingham Extended ADL Index (NEADL [20])) and cognitive function (Informant Questionnaire on Cognitive Decline in the Elderly (IQCODE [21])). Estimated height was derived using knee-heel length [22] and the patients were weighed using a chair scale. Mobilization after surgery was used as a process measure, recorded on day two post-surgery from case notes and observations. From September 2011, mobility was recorded with the activPAL™ body-worn sensor system [23]. The sensor was attached on the anterior aspect of the non-affected thigh as soon as possible after surgery and worn until discharge.

All patients were screened once daily for delirium using the Confusion Assessment Method (CAM) [24] preoperatively and until the fifth postoperative day (all) or until discharge (delirious patients). The study geriatrician or a study nurse completed all the assessments. If the nurse was unsure about the diagnosis, the study geriatrician was consulted. The CAM score was based on information from nurses, close relatives and hospital records related to the preceding 24 hours, in combination with a 10 to 30 minute interview with the patient. Tests of cognition, attention and alertness included the digit span test (forward and backward), orientation and delayed recall (from the Memorial Delirium Assessment Scale (MDAS) [25]). Delirium severity was measured with MDAS. Patients were assessed regularly on weekdays, but staff members who had been working during weekends were interviewed every Monday, and the case notes scrutinized in order to ascertain potential episodes of delirium. The mean number of delirium assessments during the stay was 5.7 (SD 2.7).

Follow up visits were carried out four and twelve months after surgery (with a time window of ± three weeks) by study nurses blind to allocation and to all clinical data during the original hospital stay. The patients were assessed in their current place of residence. Each visit typically lasted for two to three hours, and the evaluators started the assessment with the cognitive tests of the primary outcome.

At each follow-up visit, proxies were interviewed regarding physical (ADL) and cognitive function, using the same scoring systems as during the index stay. Mobility at the follow-up visits was assessed with the short physical performance battery (SPPB) [26]. Weight at follow-up was assessed using a standing scale that was calibrated to the chair scale used during hospital stay. Patients and proxies were asked about any hospital readmissions since surgery.

One specialist in geriatric medicine (TBW) and one specialist in old age psychiatry (KE) independently assessed whether the patients fulfilled the International Classification of Diseases, version 10 (ICD-10) criteria for dementia at baseline and 12 months after surgery. The assessors had access to all clinical data, but were blinded to allocation and delirium status during hospital stay. The inter-rater agreement upon the dementia diagnosis was satisfactory (kappa 0.87 at baseline and 0.83 at 12 months); disagreements were resolved through discussion.

### Primary outcome

The primary outcome was cognitive function four months after surgery, which was expected to show a wide range of severity from severe dementia to no cognitive impairment. To be able to measure differences in both the higher and the lower spectrum of cognitive function, we combined two scales:

– The 10 words test from the Consortium to Establish a Registry for Alzheimer’s disease battery (CERAD) [27]. In this memory test patients are asked to recall 10 words after having them presented orally or visually. We used the immediate and delayed recall tasks of the test. This test is shown to be sensitive for memory changes in persons with good cognitive functioning [28].

– The Clinical Dementia Rating scale (CDR [29]). CDR is based on information from the best available sources as a combination of patient and proxy information and is sensitive for cognitive changes in patients with dementia. We used the ‘sum of boxes’ scoring adding up to a sum score ranging from zero (no dementia symptoms) to 18 (severe dementia). In most studies the sum score is shown to correlate highly with the original categorical score of zero to three [30].

To construct the combined outcome measure, we normalized these scales into a 0 to 100 scoring (CDR had to be reversed since it is scaled in the opposite direction). The CDR carried a 50% weighting, and the immediate and delayed recall parts of the 10 word test each contributed 25% in the combined measure. Thus, a higher score on the primary outcome indicated better cognitive performance.

### Secondary outcomes

Secondary outcomes included preoperative delirium, delirium severity, length of stay, mortality, mobility, place of residence, ADL function and weight changes at the follow up controls. CDR and the 10 words test were analyzed separately, in addition to other measures of cognition (Mini-mental state examination (MMSE) [31], clock drawing test [32], IQCODE).

### Statistical analyses

No pre-trial data were available to carry out precise power estimates. Based upon previous experience with the CDR, we judged 300 patients to be sufficient to detect clinically meaningful differences [30]. As 20% of hip fracture patients can be expected to die within four months of surgery, we aimed to randomize 370 patients. Recruitment ended after randomization of 332 patients due to resource constraints.

A statistical analysis plan (SAP) was developed (and published online) prior to un-blinding of the data [33]. The primary analysis was carried out blind to allocation by the study statistician (ES).

**Figure 1:**
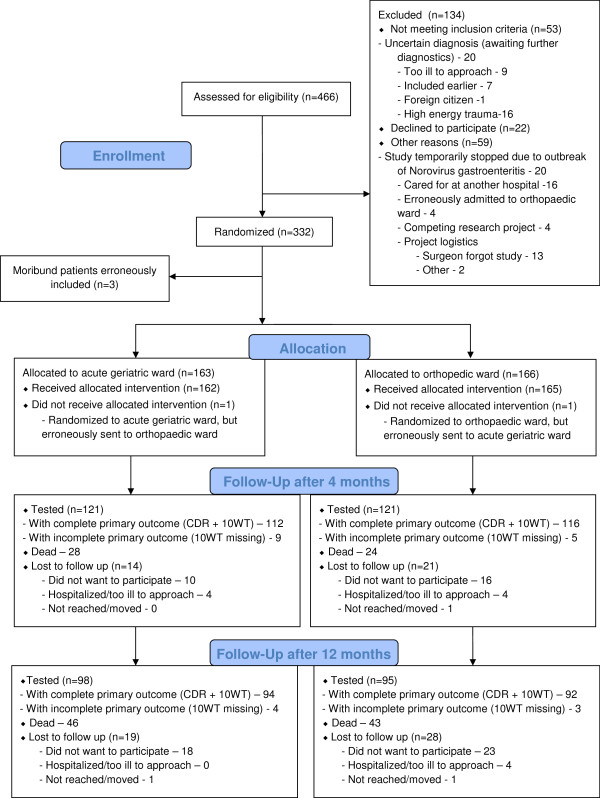
**CONSORT flow diagram.** CDR, Clinical Dementia Rating; 10 WT, 10 word test (from CERAD, Consortium to Establish a Registry for Alzheimer’s Disease).

The primary analysis was carried out as a modified intention-to-treat analysis including patients with CDR and a complete 10-word test at the four-month control. Two patients were sent to the ward opposite to randomization allocation, and these patients were analyzed according to the group in which they were treated (Figure 1). Three moribund patients (two randomized to the acute geriatric ward and one to the orthopedic ward) were recruited in error, and were excluded from the primary analysis.

The primary outcome was not normally distributed but the sample size was large and parametric methods could therefore be applied. To adjust for any inequality in the distribution of important prognostic variables between the intervention and control group, we performed a linear regression with the primary outcome as the dependent variable, and variables with known or believed influence on the outcome were included in the model in a stepwise manner, in addition to the randomization group. If their introduction to the model changed the effect estimate for the randomization variable by 10% or more, they were included in the final model. Variables were removed by stepwise backwards elimination until the final model was reached. Age (negatively skewed) and waiting time to surgery (positively skewed) had non-normal distributions, and were squared and log transformed, respectively, to achieve better fit of the model. Secondary outcomes were analyzed by the Mann–Whitney test, t-tests and Chi-square tests depending on data distribution. Pre-planned subgroup analyses were carried out in patients admitted from nursing homes, and in patients with and without pre-fracture dementia.

All statistical analyses were performed using IBM SPSS Statistics version 20, except for median differences and corresponding 95% confidence intervals that were estimated by the Hodges Lehmann estimator using StatXact 8.0.

### Sensitivity analyses

As a sensitivity analysis we analyzed the primary outcome with the non-parametric Mann–Whitney test. We also carried out sensitivity analyses including the three moribund patients who were erroneously recruited, and a strict intention to treat analysis with all patients analyzed according to allocation. Missing values for the primary outcome were imputed in different ways in order to explore their potential influence on the results:

– if a patient had the combined outcome available after twelve but not four months, those values were imputed in the four-month dataset (ten patients).

– imputation of the worst possible score for all patients who had died.

– imputation of the worst possible score for all missing patients.

– imputation of the mean score for the randomization group the patient belonged to for all missing patients.

### Ethical considerations

The study was conducted in accordance with the Declaration of Helsinki. Informed consent was obtained from the patients or substitute decision-makers if patients did not have capacity to consent. The study was approved by the Regional Committee for Ethics in Medical Research in Norway (REK S-09169a) and the Data Protection Officer at Oslo University Hospital (Ref. 1361).

## Results

Between 17 September 2009 and 5 January 2012, 446 patients were assessed for eligibility and 332 were included (Figure 1). Non-included patients were younger than included patients (median 81 versus 85 years; *P* ≤0.001) and more were men (35.3% versus 25.1%, *P* = 0.01). Half of the included patients at baseline were considered to have dementia, and one third were living in nursing homes. Patients randomized to the intervention group and the control group were well matched in all important baseline variables (Table 2). In total, 35 patients (11%) were lost to follow up at four months, 14 from the intervention group and 21 from the control group (*P* = 0.23). Of patients lost to the four month follow up, only 2 (7%) were living in a nursing home before the fracture, compared to 73 (30%) patients who were followed-up (*P* = 0.002). Patients lost to follow up were younger (median age 83 versus 85, *P* = 0.19) and fewer were considered to have dementia before the fracture (12/35 (34%) versus 112/242 (46%), *P* = 0.18); however, these differences were not significant. The final twelve month follow up was completed in December 2012.

**Table 2:** Baseline characteristics

**Characteristics**	**Acute geriatric ward (number = 163)**	**Orthopedic ward (number = 166)**
Age, median (range)	84 (55 to 99)	85 (46 to 101)
Male (%)	42 (26)	38 (23)
IQCODE >3.44 (%)^a^	93 (58)	91 (58)
Dementia, expert opinion (%)^b^	80 (49)	82 (49)
BADL, median (IQR)^c^	18 (13 to 20)	18 (15 to 20)
NEADL, median (IQR)^d^	28 (9 to 52)	30.5 (12 to 52)
APACHE II score, mean (SD)	9.5 (2.8)	9.3 (2.7)
CCI, median (IQR)	1 (0 to 2)	1 (0 to 2)
Number of medications used regularly, median (IQR)	5 (2 to 7)	4 (2 to 6)
BMI, mean (SD)^e^	24.4 (4)	24.4 (4.6)
Living in an institution (%)	52 (32)	50 (30)
Type of fracture (%):		
- Femoral neck	98 (60)	97 (58)
- Intertrochanteric	64 (39)	67 (40)
- Subtrochanteric	1 (1)	2 (1)
Type of surgery (%):		
- Hemiarthroplasty	74 (45)	71 (43)
- Osteosynthesis	88 (54)	91 (55)
- Total hip replacement	0 (0)	1(1)
- Girdlestone	1 (1)	0 (0)
- Not operated	0 (0)	3 (2)
Type of anesthesia (%)		
- General	8 (5)	14 (9)
- Spinal	147 (94)	143 (91)
- Epidural	2 (1)	0 (0)
Injury occurred indoors (%)	136 (84)	139 (84)

^a^IQCODE was missing in two patients from the acute geriatric ward and in eight patients from the orthopedic ward; ^b^based upon consensus in an expert panel (TBW and KE); ^c^Barthel ADL was missing in one patient from the acute geriatric ward and three patients from the orthopedic ward; ^d^NEADL was missing in four patients from the acute geriatric ward and in two patients from the orthopedic ward; ^e^BMI was missing in 30 patients from the acute geriatric ward and in 69 patients from the orthopedic ward. APACHE II, Acute Physiology and Chronic Health Evaluation II; BADL, Barthel Activities of Daily Living; BMI, body mass index; CCI, Charlson Comorbidity Index score; IQCODE, Informant Questionnaire on Cognitive Decline in the Elderly; IQR, interquartile range; NEADL, Nottingham Extended ADL Index.

### Impact of intervention during hospital stay

There was no difference in delirium rates between the intervention and control groups (49% versus 53%, *P* = 0.51) (Table 3). There was also no difference in delirium duration (median three versus four days, *P* = 0.85) or delirium severity measured with MDAS (median 21.5 versus 20, *P* = 0.44). Fewer patients treated in the acute geriatric ward were discharged with ongoing delirium (15% versus 26%, *P* = 0.01).

**Table 3:** Impact of intervention during hospital stay

**Variable**	**Acute geriatric ward (number = 163)**	**Orthopedic ward (number = 166)**	***P*-value**
Delirium any time during hospital stay (%)^a^	80 (49)	86 (53)	0.51
Pre-operative delirium (%)^b^	47 (31)	50 (35)	0.41
Delirium severity MDAS, median (IQR)^c^	21.5 (15.3 to 25)	20 (13.8 to 26)	0.44
Delirium duration in days, median (IQR)^d^	3 (2 to 7)	4 (2 to 6)	0.85
Discharged with ongoing delirium (%)	24 (15)	43 (26)	0.01
Waiting time for surgery in hours, median (IQR)^e^	26.2 (15.9 to 42.7)	23.9 (16.5 to 38.1)	0.54
Length of stay in days, median (IQR)	11 (8 to 15)	8 (4.8 to 11)	≤ 0.001
Medical complications, any	72 (44)	76 (46)	0.82
- Cardiac complications	22 (14)	19 (11)	0.58
- Cerebral complications	2 (1)	0 (0)	0.25
- Thrombo-embolic complications	2 (1)	0 (0)	0.25
- Pulmonary complications	21 (13)	13 (8)	0.15
- Renal failure	6 (4)	2 (1)	0.18
- Urinary tract infections	26 (16)	41 (25)	0.05
- Pressure ulcer	3 (2)	8 (5)	0.22
- Gastro-intestinal complications	5 (3)	4 (2)	0.75
Surgical complications, any	4 (3)	6 (4)	0.75
- surgical site infection	1 (1)	1 (1)	1
- wound problem	2 (1)	4 (2)	0.69
- osteosynthesis failure	1 (1)	0 (0)	1
- dislocation of prosthesis	0 (0)	1 (1)	0.5
Fall (%)	14 (9)	11 (7)	0.5
In-hospital mortality (%)	6 (4)	3(2)	0.21
Mobilized out of bed the second day after surgery (%)^f^	139 (86)	119 (80)	0.13
Time mobilized in standing or stepping position the first five days after surgery in minutes, median (IQR)^g^	29.3 (10.8 to 42.7)	16.8 (4.3 to 68.2)	0.24

^a^Delirium status defined by CAM. CAM was missing in four patients from the orthopedic ward; ^b^preoperative delirium status unknown in nine patients from the acute geriatric ward and in 23 patients from the orthopedic ward; ^c^highest MDAS in patients with delirium. MDAS was missing in four patients in the acute geriatric ward and in eight patients from the orthopedic ward; ^d^number of days from first to last positive CAM; ^e^time from admission to start of anesthesia. Three patients from the orthopedic ward did not undergo surgery; ^f^missing in two patients from the acute geriatric ward and in 17 patients from the orthopedic ward; ^g^masured with activPAL™ in 22 patients from the acute geriatric ward and in 24 patients from the orthopedic ward. CAM, Confusion Assessment Method; IQR, interquartile range; MDAS, Memorial Delirium Assessment Scale.

The median length of stay was three days longer in the intervention group (median eleven versus eight days, *P* ≤0.001). Patients in the intervention group had a longer waiting time for surgery, but this difference was not statistically significant (median 26 versus 24 hours, *P* = 0.54).

There was a trend to greater mobilization in the intervention group on the second day after surgery (86% versus 80%). In 46 patients, mobilization after surgery was assessed with activPAL™ activity sensors. During the first five days after surgery, the patients were mobilized for a longer time in the standing or stepping position in the intervention group (median 29 minutes versus 17 minutes).

### Primary outcome - cognitive function four months after surgery

The primary outcome could be computed in 228 patients and there was no significant difference between patients treated in the acute geriatric ward and the orthopedic ward after four months (mean 54.7 versus 52.9, 95% confidence interval for the difference -5.9 to 9.5; *P* = 0.65) (Table 4). There was also no difference in the combined outcome after twelve months (mean 51.0 versus 49.1, 95% confidence interval for the difference -7.7 to 11.4; *P* = 0.69). The Mann–Whitney test gave essentially the same results as the t-test at four and twelve months. A linear regression with the primary outcome as the dependent variable (Table 5) identified four significant predictors associated with poorer score: if the patient was admitted from a nursing home, IQCODE at baseline above 3.44, older age, and delirium during the hospital stay.

**Table 4:** Impact of intervention four and twelve months after surgery

	**Four months follow up**			**Twelve months follow up**		
**Outcome**	**Acute geriatric ward (n = 121)**	**Orthopedic ward (n = 121)**	***P*-value**	**Acute geriatric ward (n = 98)**	**Orthopedic ward (n = 95)**	***P*-value**
Primary outcome, mean (SD)^a^	54.7 (30.3)	52.9 (29.1)	0.65	51.0 (33.4)	49.1 (32.3)	0.69
CERAD 10 word test, median (IQR)						
- immediate recall,	12.5 (6 to 17)	11.5 (5.3 to 18)	0.77	11.5 (5 to 18)	11 (5 to 17.8)	0.89
- delayed recall	3 (0 to 6)	2 (0 to 5)	0.35	3 (0 to 6)	2.5 (0 to 5)	0.41
- recognition	18 (13.4 to 19)	17.5 (13 to 19.8)	0.93	17 (11 to 20)	17 (12 to 20)	0.93
CDR sum of boxes, median (IQR)	1.5 (0 to 9)	2.5 (0 to 9.5)	0.39	1.75 (0 to 14)	2.5 (0 to 14)	0.52
MMSE, median (IQR)^b^	24 (16 to 28)	23 (16 to 27)	0.28	24 (16.3 to 27)	22 (13.3 to 26)	0.34
Approved clock drawing test (%)^c^	48 (49)	42 (40)	0.20	39 (46)	28 (35)	0.12
NEADL, median (IQR)^d^	26.5 (7.8 to 50.3)	22 (9 to 46.5)	0.85	25 (8.8 to 51)	18 (10 to 47)	0.65
BADL, median (IQR)^e^	17 (10 to 20)	16 (12 to 20)	0.80	17 (9.5 to 19)	16 (11 to 19)	0.44
SPPB, median (IQR)^f^	4 (1 to 8)	3 (1 to 6)	0.13	3 (1 to 7)	3 (1 to 6)	0.14
IQCODE, median (IQR)^g^	3 (3 to 3.25)	3 (3 to 3.19)	0.74	3.69 (3 to 5)	3.75 (3.13 to 4.94)	0.45
Weight change from index stay in kg, mean (SD)^h^	- 3.4 (4.3)	- 4.4 (5.0)	0.25	- 2.4 (6.3)	- 3.4 (7)	0.43
New nursing home admissions (%)	19 (16)	18 (15)	0.86	16 (16)	18 (19)	0.63
Incident dementia^i^				7 (7)	3 (3)	0.33
Re-admissions	21 (17)	21 (17)	0.95	32 (33)	33 (35)	0.76

^a^To construct the primary outcome, we normalized CDR and the 10 word test from CERAD into a 0 to 100 scoring (CDR had to be reversed since it is scaled in the opposite direction). CDR weighed 50% and the immediate and delayed recall parts of the 10 word test weighed 25% each in the combined measure. The primary outcome was missing in nine patients from the acute geriatric ward and five patients from the orthopedic ward at four months and in four patients from the acute geriatric and three from the orthopedic ward at twelve months; ^b^MMSE was missing in 11 patients from the acute geriatric ward and in nine patients from the orthopedic ward at the four-month control and in six and three patients, respectively, at the 12-months control; ^c^ ≥ 4 points. Clock drawing test was missing in 23 patients from the acute geriatric ward and in 16 patients from the orthopedic ward at the four-month control and 14 and 14 patients, respectively, at the 12-month control; ^d^NEADL was missing in seven patients from the acute geriatric ward and in eight from the orthopedic ward at the four-month control and in two patients from the orthopedic ward from the 12-month control; ^e^Barthel ADL was missing in two patients from the acute geriatric ward and in one from the orthopedic ward at four months and in one and two patients, respectively, at the 12-month control; ^f^SPPB was missing in seven patients from the acute geriatric ward and two from the orthopedic ward at four months and in five and three patients, respectively, at the 12-month control; ^g^a modified version of IQCODE was used at the four-month control; instead of asking for changes in the last 10 years, we asked for changes since just before the hip fracture. At the 12-month control we used the regular IQCODE. This was missing in two patients from the acute geriatric ward and three from the orthopedic ward at the four-month control and in three and four patients, respectively, at the 12-month control; ^h^weight was missing in 33 patients from the acute geriatric ward and 29 patients from the orthopedic ward at the four-month control and in 19 and 22 patients, respectively, at the 12-month control; ^i^based upon consensus in an expert panel (TBW and KE). BADL, Barthel Activities of Daily Living; CDR, The Clinical Dementia Rating scale; CERAD, Consortium to Establish a Registry for Alzheimer’s Disease; IQCODE, Informant Questionnaire on Cognitive Decline in the Elderly; IQR, interquartile range; MMSE, Mini Mental State Examination; NEADL, Nottingham Extended ADL Index; SD, standard deviation; SPPB, Short Physcial Performance Battery.

**Table 5:** Multiple linear regression model with the primary outcome at the four-month follow up control as the dependent variable (number = 228)

**Variable**	**Unadjusted coefficients (95% CI)**	***P*-value**	**Adjusted coefficients (95% CI)**	***P*-value**
Randomization group (reference: orthopedic ward)	1.8 (-5.9 to 9.5)	0.65	-2.5 (-7.1 to 2.2)	0.29
Admitted from nursing home	-44.5 (-50.8 to -38.2)	≤0.001	-25.0 (-31.1 to -18.8)	≤0.001
Age^a^	-0.006 (-0.009 to -0.004 )	≤0.001	-0.002 (-0.003 to 0.000)	0.03
Gender (reference: male)	6.94 (-2.4 to 16.3)	0.14		
Delirium during hospital stay^b^	-31.7 (-38.3 to -25.0)	≤0.001	-11.7 (-17.1 to -6.3)	≤0.001
Number of years of higher education^c^	2.17 (-0.19 to 4.16)	0.03		
IQCODE >3.44^d^	-42.3 (-47.9 to 36.7)	≤0.001	-23.4 (-29.4 to -17.5)	≤0.001
Preoperative waiting time^e^	1.93 (-4.0 to 7.85)	0.52		
APACHE II	-0.78 (-2.2 to 0.66)	0.29		

R = 0.82. ^a^Age squared; ^b^number = 226; ^c^number = 203; ^d^IQCODE obtained during hospital stay (number = 222); ^e^natural logarithm of preoperative waiting time. APACHE II, Acute Physiology and Chronic Health Evaluation II; IQCODE, Informant Questionnaire on Cognitive Decline in the Elderly.

### Secondary outcomes and subgroup analyses

Patients treated in the acute geriatric ward performed better on all cognitive measures; CERAD immediate recall (median 12.5 versus 11.5) and delayed recall (median 3 versus 2) , approved clock drawing test (49% versus 40%), MMSE (median 24 versus 23) and CDR (1.5 versus 2.5). They also had better ADL function measured with the BADL Index (median 17 versus 16) and NEADL (median 26.5 versus 22). None of these differences were, however, statistically significant.

Patients randomized to the acute geriatric ward had better mobility four months after surgery, measured with SPPB (median 4 versus 3, 95% confidence interval for the median difference 0 to 2; *P* = 0.13) (Table 4). This difference was statistically significant in the pre-specified subgroup analysis restricted to patients living in their own home before the fracture (median 6 versus 4, 95% confidence interval for the median difference 0 to 2; *P* = 0.04). Subgroup analyses stratified according to pre-fracture dementia status and nursing home residence gave no other significant differences, except that patients from nursing homes randomized to intervention were more often mobilized the second day after surgery (Additional files 1, 2 and 3).

Twenty-eight (17%) patients treated in the acute geriatric ward and 24 (15%) treated in the orthopedic ward were dead four months after the surgery (*P* = 0.50). In both groups, 21% of the patients were readmitted during the first four months after surgery. The results at the 12-month follow up were similar to those after four months.

### Sensitivity analyses

Several sensitivity analyses were performed and they showed no substantial differences from the primary analysis.

## Discussion

In this randomized controlled trial of patients with hip fracture, we found no evidence that cognitive function four months after surgery was improved in patients treated pre- and postoperatively in an acute geriatric ward, compared to usual care in an orthopedic ward. Delirium rates were equally high in both groups. There was, however, a trend that the intervention had a positive effect on mobility.

### Strength and weaknesses

The main strength of this study was the randomized controlled design with blinded outcome assessments. Also, the inclusion of process measures, such as objective mobilization scores, confirms that the intervention was being delivered as intended. The inclusion of patients from nursing homes and those with dementia enhances generalizability as such patients are frequently excluded from trials [34]. On the other hand, nursing home patients are so frail and cognitively impaired that they may be unlikely to benefit from the intervention. To assess the efficacy in such patients, other outcomes than those we chose might be more feasible [35]. The combined outcome measure was designed to measure cognition in patients representing a broad spectrum of cognitive function and was based upon well-validated components. However, the scale combination has not been validated and, thus, we cannot be sure that it was sensitive to the intervention. As with all service evaluations, blinding of assessments during hospital stay was impossible and may have introduced bias.

Inclusion was terminated before the intended sample size aim was reached. However, as there were few differences between the groups in any of the secondary cognitive outcomes, and sub-group analyses also failed to show any substantial differences, it is reasonable to conclude that this intervention had no effect on cognition.

### Comparison with other studies

The impact of orthogeriatric intervention on long-term cognitive function has not previously been assessed. Several studies have demonstrated that orthogeriatric care can prevent delirium in hip fracture patients. A recently published non-randomized controlled trial from Belgium [15] showed that an intervention provided by an inpatient geriatric consultation team was effective in reducing the incidence of delirium (37.2% versus 53.2%, *P* = 0.04), in keeping with a similar American RCT [16]. In both the American and the Belgian studies, all patients received standard treatment from the orthopedic team, whereas in our model orthopedic treatment (besides surgery) was limited to consultation service. A possible explanation for the lack of effect of our model could, therefore, be limited access to orthopedic expertise.

Few studies have compared pre- and postoperative intervention provided in a geriatric ward with usual care in an orthopedic ward. In comparison with usual care, such models have shown promising results, but cognition has seldom been assessed [5,6]. To our knowledge, the only RCT evaluating a geriatrician-led fracture service (were geriatricians have the primary responsibility for the patients) is a Swedish study [36]. Although no preoperative intervention was included, the study showed that significantly more patients allocated to intervention regained independence in personal ADL performance at four and twelve months after surgery. The model was also effective in preventing postoperative delirium and reducing delirium duration [14]. In spite of the fact that we also included preoperative intervention, we were not able to prevent delirium. A likely explanation is that usual care was better in our study since the delirium rates both in the intervention and the control group were lower than in the Swedish study. The orthopedic ward in our study provided a short waiting time for surgery, similar staffing as in the geriatric ward, personnel with earlier experience with orthogeriatric models and delirium prevention, physiotherapy for most hip fracture patients, and an integrated post-operative care unit.

Orthogeriatric intervention is often reported to reduce waiting time for surgery (see Liem [37] for an overview). In our study, however, the waiting time for surgery was two hours longer in the intervention group. Both the intervention (26 hours) and the control group (24 hours) waited, however, for a short time compared to other orthogeriatric studies reporting a waiting time of two to three days and even longer [38-42], indicating that the control group received a good quality service.

Mobility has been assessed in several studies, but mostly by questionnaire. Some, but not all, studies have found that orthogeriatric services provide better mobility [36,40,43,44]. In our study there was an overall trend that patients treated in the intervention group performed better at SPPB four months after surgery, and the difference was statistically significant in those living in their own homes before surgery. A difference on SPPB of 0.5 is considered clinically meaningful, and the effect seen in our study (six versus four points) is likely to be important and should be further explored in future studies.

### Interpretation of the results

Despite our comprehensive intervention, the effect on the primary outcome was limited. There are several possible explanations for this. First, the choice of cognitive function as the primary outcome may have been too ambitious. For the intervention to be effective in this regard, two pre-suppositions had to be true. First, the orthogeriatric intervention had to be effective in reducing delirium. However, our intervention failed to prevent delirium or reduce delirium severity. This might be explained by the good quality of usual care at the orthopedic ward in our hospital, combined with sub-optimal circumstances in an often over-crowded acute geriatric ward.

Secondly, the primary outcome assumes that delirium lies on the causal pathway towards the development of dementia. Since delirium usually occurs in relation to acute illness, it is challenging to design studies that can address this question, but some evidence exits suggesting that delirium is associated with long term cognitive decline [13,45,46]. Our study is in keeping with this; the regression analysis showed that delirium was associated with a poorer score on the primary outcome, also when adjusting for potential confounders.

The study may have influenced treatment in the control group. The patients in the orthopedic ward were assessed daily, and in order to make a precise delirium diagnosis personnel in the orthopedic ward were interviewed regarding the patients cognitive status. This inevitably raised the awareness of delirium in the orthopedic ward.

## Conclusions

This randomized controlled trial of hip fracture patients found no evidence that cognitive function four months after surgery was improved in patients treated with pre- and postoperative orthogeriatric care provided in an acute geriatric ward, compared to usual care in an orthopedic ward. The intervention had a positive effect on mobility in patients not admitted from nursing homes. Delirium had a strong negative impact on long-term cognitive performance, and delirium prevention and treatment should be given high priority in orthogeriatric care. For further orthogeriatric improvements, we recommend a model with stronger integration of orthopedic and geriatric input than we achieved, in line with recommendations from recent reviews [5,7].

## Competing interests

The authors declare that they have no competing interests.

## Authors' contributions

TBW initiated the study, led the work on the study design and was involved in analyzing and interpreting the data. TBW is the manuscript’s guarantor. LOW had the daily responsibility for running the study and collecting data. LOW was also involved in planning of the study, has analyzed and interpreted the data and drafted the manuscript. ACT had particular responsibility for collecting nutritional data and was involved in planning the study. FF and GH had the primary responsibility to remind and motivate the orthopedic surgeons to include patients in the study. KE participated in all aspects of the planning, in particular regarding the cognitive outcomes. ES carried out the randomization procedure and was extensively involved in the statistical planning and analyses. Together with LOW and TBW, she wrote the statistical analysis plan. FF, VJ, IS, JR, ES and SC all made important contributions to the planning of the study and writing the protocol. All authors participated in critical revision of the article for intellectual content. All authors read and approved the final version of the manuscript.

## Pre-publication history

The pre-publication history for this paper can be accessed here:

http://www.biomedcentral.com/1741-7015/12/63/prepub

## Supplementary Material

Additional file 1 **Impact of intervention during hospital stay.** Patients stratified according to prefracture residential status (1a) and dementia status (1b). Click here for file

Additional file 2 **Impact of intervention four months after surgery.** Patients stratified according to prefracture residential status (2a) and dementia status (2b). Click here for file

Additional file 3 **Impact of intervention 12 months after surgery.** Patients stratified according to prefracture residential status (3a) and dementia status (3b). Click here for file
