# Supplementary material for: The effect of a pre- and postoperative orthogeriatric service on cognitive function in patients with hip fracture: randomized controlled trial (Oslo Orthogeriatric Trial)
Source: BMC Med. 2014 Apr 15;12:63. doi: 10.1186/1741-7015-12-63 (PMC4022270; doi:10.1186/1741-7015-12-63)
Supplement: Additional file 1 — Impact of intervention during hospital stay. Patients stratified according to prefracture residential status (1a) and dementia status (1b). [file 1741-7015-12-63-S1.docx]

**Additional file 1.** Impact of intervention during hospital stay. Patients stratified according to prefracture residential status (1a) and dementia status (1b).

1 a)

|  | Home-dwelling patients (n=227) | | | Patients from nursing homes (n=102) | | |
| --- | --- | --- | --- | --- | --- | --- |
|  | Acute geriatric ward (n=111) | Orthopaedic ward (n=116) | P-value | Acute geriatric ward (n=52) | Orthopaedic ward (n=50) | P-value |
| Delirium any time during hospital stay (%)^a^ | 40 (36) | 49 (42) | 0.31 | 40 (77) | 37 (79) | 0.83 |
| Pre-operative delirium (%)^b^ | 23 (21) | 24 (24) | 0.64 | 24 (52) | 26 (61) | 0.43 |
| Delirium severity MDAS, median (IQR)^c^ | 17 (11 - 23) | 17 (11.8 - 21) | 0.87 | 24 (20 - 26.5) | 23.5 (18 - 26) | 0.57 |
| Delirium duration in days, median (IQR)^d^ | 3.5 (2 - 8.8) | 3 (1.5 - 6) | 0.25 | 3 (2 - 5) | 4 (2 - 5.5) | 0.35 |
| Discharged with ongoing delirium (%) | 6 (5) | 15 (13) | 0.08 | 18 (35) | 28 (56) | 0.006 |
| Waiting time for surgery in hours, median (IQR)^e^ | 26.9 (17.9 - 42.8) | 23.8 (17.7 - 38.5) | 0.47 | 23.7 (15.7 - 42-1) | 24.6 (13.7 - 37.5) | 0.86 |
| Length of stay in days, median (IQR) | 12 (10 - 16) | 9 (8 - 11) | ≤ 0.001 | 8 (5 - 12) | 4 (3 - 7) | ≤ 0.001 |
| In-hospital mortality (%) | 2 (2) | 3 (3) | 1.0 | 4 (8) | 0 (0) | 0.12 |
| Mobilised out of bed the second day after surgery (%)^f^ | 101 (91) | 95 (82) | 0.73 | 38 (73) | 24 (48) | 0.04 |
| Time mobilised in standing or stepping position the first five days after surgery in minutes, median (IQR)^g^ | 37.3 (25.1 - 79.6) | 20.1 (4.8 - 69.6) | 0.32 | 9.6 (1.8 - 13.7) | 4.1 (0.3 - 5.5) | 0.13 |

^a^ Delirium status defined by CAM. Home-dwelling patients: CAM was missing in one patient from orthopaedic ward. Nursing home patients: CAM was missing in three patients from the orthopaedic ward

^b^ Home-dwelling patients: preoperative delirium status unknown in three patients from the acute geriatric ward and in 16 patients from the orthopaedic ward. Nursing home patients: preoperative delirium status unknown in six patients from the acute geriatric ward and in seven patients from the orthopaedic ward.

^c^ Highest MDAS in patients with delirium. Home-dwelling patients: MDAS was missing in one patient from the acute geriatric ward and in seven from the orthopaedic ward. Nursing home patients: MDAS was missing in three patients from the acute geriatric ward and in one patient from the orthopaedic ward.

^d^ Number of days from first to last positive CAM

^e^ Time from admission to start of anaesthesia. Home-dwelling patients: Three patients from the orthopaedic ward did not undergo surgery.

^f^ Home-dwelling patients: missing in one patient from the acute geriatric ward and in 11 patients from the orthopaedic ward. Nursing home patients: missing in one patient from the acute geriatric ward and in six patients from the orthopaedic ward.

^g^ Measured with activPAL™ from September 2011. Home-dwelling patients: n= 16 from the acute geriatric ward and n= 19 from the orthopaedic ward. Nursing home patients: n=6 from the acute geriatric ward and n=5 from the orthopaedic ward.

CAM = Confusion Assessment Method. MDAS = the Memorial Delirium Assessment Scale. IQR = Interquartile Range.

1 b)

|  | No dementia (n=167) | | | Dementia (n=162) | | |
| --- | --- | --- | --- | --- | --- | --- |
|  | Acute geriatric ward (n=83) | Orthopaedic ward (n=84) | P-value | Acute geriatric ward (n=80) | Orthopaedic ward (n=82) | P-value |
| Delirium any time during hospital stay (%)^a^ | 21 (25) | 26 (31) | 0.39 | 59 (74) | 60 (76) | 0.75 |
| Pre-operative delirium (%)^b^ | 9 (11) | 11 (15) | 0.45 | 38 (54) | 39 (57) | 0.72 |
| Delirium severity MDAS, median (IQR)^c^ | 15.5 (9.3 - 21) | 17 (9 - 22) | 0.83 | 23 (18.3 - 26) | 21 (16 - 26) | 0.25 |
| Delirium duration in days, median (IQR)^d^ | 3 (1.5 - 4.5) | 2.5 (1 - 6.5) | 0.88 | 4 (2 - 8) | 4 ( 2 - 6) | 0.92 |
| Discharged with ongoing delirium (%) | 2 (2) | 4 (5) | 0.69 | 22 (28) | 39 (48) | 0.002 |
| Waiting time for surgery in hours, median (IQR)^e^ | 26.9 (17.2 - 40.7) | 23.8 (18.4 - 36.3) | 0.67 | 24.3 (15.9 - 45.6) | 24.4 (14 - 39.8) | 0.52 |
| Length of stay in days, median (IQR) | 11 (9 - 15) | 9 (7 - 11) | ≤ 0.001 | 11 (6 - 14) | 7 (3 - 10) | ≤ 0.001 |
| In-hospital mortality (%) | 3 (4) | 1 (1) | 0.38 | 3 (4) | 2 (2) | 0.68 |
| Mobilised out of bed the second day after surgery (%)^f^ | 76 (92) | 72 (86) | 0.93 | 63 (79) | 47 (57) | 0.06 |
| Time mobilised in standing or stepping position the first five days after surgery in minutes, median (IQR)^g^ | 35.5 (22.6 - 91.2) | 38.4 (12.8 - 93.1) | 0.73 | 10.5 (2.2 - 32.9) | 5.1 (0.7 - 11.2) | 0.32 |

^a^ Delirium status defined by CAM. No dementia: CAM was missing in one patient from orthopaedic ward. Dementia: CAM was missing in three patients from the orthopaedic ward

^b^ No dementia: preoperative delirium status unknown in 10 patients from the orthopaedic ward. Dementia: preoperative delirium status unknown in nine patients from the acute geriatric ward and in thirteen patients from the orthopaedic ward.

^c^ Highest MDAS in patients with delirium. No dementia: MDAS was missing in one patient from the acute geriatric ward and in three from the orthopaedic ward. Dementia: MDAS was missing in three patients from the acute geriatric ward and in five patients from the orthopaedic ward.

^d^ Number of days from first to last positive CAM

^e^ Time from admission to start of anaesthesia. Dementia: Three patients from the orthopaedic ward did not undergo surgery.

^f^ Home-dwelling patients: missing in one patient from the acute geriatric ward and in 11 patients from the orthopaedic ward. Nursing home patients: missing in one patient from the acute geriatric ward and in six patients from the orthopaedic ward.

^g^ Measured with activPAL™ from September 2011. No dementia: n= 14 from the acute geriatric ward and n= 14 from the orthopaedic ward. Dementia: n=8 from the acute geriatric ward and n=10 from the orthopaedic ward.

CAM = Confusion Assessment Method. MDAS = the Memorial Delirium Assessment Scale. IQR = Interquartile Range.
