# Supplementary material for: The effect of a pre- and postoperative orthogeriatric service on cognitive function in patients with hip fracture: randomized controlled trial (Oslo Orthogeriatric Trial)
Source: BMC Med. 2014 Apr 15;12:63. doi: 10.1186/1741-7015-12-63 (PMC4022270; doi:10.1186/1741-7015-12-63)
Supplement: Additional file 3 — Impact of intervention 12 months after surgery. Patients stratified according to prefracture residential status (3a) and dementia status (3b). [file 1741-7015-12-63-S3.docx]

Additional file 3. Impact of intervention 12 months after surgery. Patients stratified according to prefracture residential status (3a) and dementia status (3b).

3a)

|  | Home-dwelling patients (n=139) | | | Patients from nursing homes (n=54) | | |
| --- | --- | --- | --- | --- | --- | --- |
|  | Acute geriatric ward (n=72) | Orthopaedic ward (n=67) | P-value | Acute geriatric ward (n=26) | Orthopaedic ward (n=28) | P-value |
| Primary endpoint , mean (SD)^a^ | 64.4 (26.1) | 64.5 (24.5) | 0.98 | 13.9 (21.2) | 11.9 (12.0) | 0.67 |
| CERAD 10 word test, median (IQR)^b^  - immediate recall,  - delayed recall  - recognition | 15 (10 - 19)  4 (2 - 7)  18 (16 - 20) | 15 (10.5 - 23)  4 (1.5 - 6.5)  18 (15 - 20) | 0.77  0.66  0.98 | 0 (0 - 7)  0 (0 - 0)  10 (0 - 14.5) | 3 (0 - 5)  0 (0 - 0)  11 (0 - 13) | 0.72  0.50  0.65 |
| CDR sum of boxes, median (IQR) | 0.8 (0 - 3) | 1.5 (0 - 3.5) | 0.53 | 18 (11.8 - 18) | 16 (11.3 - 18) | 0.41 |
| MMSE, median (IQR)^c^ | 25.5 (21 - 28) | 25.0 (21 - 28) | 0.64 | 1.5 (0 - 15.0) | 8 (0 - 15.0) | 0.35 |
| Approved clock drawing test (%)^d^ | 38 (59) | 27 (49) | 0.26 | 1 (5) | 1 (4) | 1.0 |
| NEADL, median (IQR)^e^ | 39.5 (19.3 - 53.8) | 35.0 (16.0 - 53.0) | 0.35 | 4.0 (2.8 - 8.3) | 8.0 (3.5 - 14.5) | 0.06 |
| BADL, median (IQR)^f^ | 19.0 (16.0 - 20.0) | 18.0 (14.8 - 19.3) | 0.25 | 8.0 (4 - 11.5) | 9.0 (4.0 - 14.0) | 0.38 |
| Died (%) | 21 (19) | 22 (19) | 0.99 | 25 (48) | 21 (42) | 0.54 |
| SPPB, median (IQR)^g^ | 5 (3 - 8) | 4 (1 - 7.3) | 0.25 | 0 (0 - 2) | 0.5 (0 - 2) | 0.94 |
| IQCODE, median (IQR)^h^ | 3.25 (3.00 - 4.13) | 3.31 (3.00 - 4.32) | 0.46 | 5.00 (4.92 - 5-00) | 5.00 (4.88 - 5.00) | 0.52 |
| Weight change from index stay in kg, mean (SD)^i^ | - 2.0 (5.4) | - 3.1 (6.8) | 0.36 | - 4.7 (10.7) | - 4.5 (8.0) | 0.96 |
| New nursing home admissions (%) | 16 (22) | 18 (27) | 0.53 |  |  |  |
| Incident dementia^j^ | 7 (10) | 3 (5) | 0.33 | 0 (0) | 0 (0) |  |
| Re-admissions | 20 (30) | 21 (33) | 0.67 | 6 (25) | 1 (4) | 0.10 |

^a^ To construct the primary endpoint, we normalised CDR and the 10 word test from CERAD into a 0 - 100 scoring (CDR had to be reversed since it is scaled in the opposite direction). CDR weighed 50 % and the immediate and delayed recall parts of the 10 word test weighed 25 % each in the combined measure. Home-dwelling patients: The primary endpoint was missing in three patients from the acute geriatric and in two patients from the orthopaedic ward. Nursing home patients: The primary endpoint was missing in one patient from the acute geriatric and in one patient from the orthopaedic ward.

^b^ Home-dwelling patients: Immediate and delayed recall missing in three patients from the acute geriatric ward and in three patients from the orthopaedic ward. Recognition missing in five patients from the acute geriatric ward and in five patients from the orthopaedic ward. Nursing home: Immediate and delayed recall missing in six patients from the acute geriatric ward and in two patients from the orthopaedic ward. Recognition missing in 14 patients from the acute geriatric ward and in 12 patients from the orthopaedic ward.

^c^ Home-dwelling patients: MMSE was missing in two patients from the acute geriatric ward and in two patients from the orthopaedic ward. Nursing home patients: MMSE was missing in four patients from the acute geriatric ward and in one patient from the orthopaedic ward.

^d^ ≥ 4 points. Home-dwelling patients: clock drawing test was missing in eight patients from the acute geriatric ward and in 12 patients from the orthopaedic ward. Nursing home patients: clock drawing test was missing in six patients from the acute geriatric ward and in two patients from the orthopaedic ward.

^e^ Nursing home patients: NEADL was missing in two patients from the orthopaedic ward.

^f^ Home-dwelling patients: Barthel ADL was missing in one patient from the orthopaedic ward. Nursing home patients: Barthel ADL was missing in one patient from the acute geriatric ward and one patient from the orthopaedic ward.

^g^ Home-dwelling patients: SPPB was missing in one patient from the orthopaedic ward. Nursing home patients: SPPB was missing in five patients from the acute geriatric ward and in two patient from the orthopaedic ward.

^h^ Home-dwelling patients: IQCODE was missing in three patients from the acute geriatric ward and in four patients from the orthopaedic ward.

^i^ Home-dwelling patients: Weight missing in 10 patients from the acute geriatric ward and 26 patients from orthopaedic ward. Nursing home patients: Weight missing in 16 patients from the acute geriatric ward and in 19 patients from orthopaedic ward.

^j^ Based upon consensus in an expert panel (TBW and KE)

SD = Standard Deviation. CERAD = Consortium to Establish a Registry for Alzheimer’s Disease. IQR = Interquartile Range. CDR = The Clinical Dementia Rating scale. MMSE = Mini Mental State Examination. BADL = Barthel Activities of Daily Living. NEADL = Nottingham Extended ADL Index. SPPB = Short Physcial Performance Battery. IQCODE = Informant Questionnaire on Cognitive Decline in the Elderly.

3b)

|  | No dementia (n=106) | | | Dementia (n=87) | | |
| --- | --- | --- | --- | --- | --- | --- |
|  | Acute geriatric ward (n=57) | Orthopaedic ward (n=49) | P-value | Acute geriatric ward (n=41) | Orthopaedic ward (n=46) | P-value |
| Primary endpoint , mean (SD)^a^ | 74.7 (15.7) | 75.9 (14.5) | 0.71 | 17.5 (20.4) | 22.3 (20.7) | 0.29 |
| CERAD 10 word test, median (IQR)^b^  - immediate recall,  - delayed recall  - recognition | 17.0 (13.0 - 21.0)  6.0 (4.0 - 7.0)  19.0 (18.0 - 20.0) | 17.5 (12.8 - 24.0)  5.0 (3.8 - 8.0)  19.0 (18.0 - 20.0) | 0.34  0.96  0.99 | 4.0 (0 - 8.0)  0 (0 - 1.0)  10.0 (0 - 16.0) | 5.0 (0 - 9.0)  0 (0 - 1)  12.0 (10.0 - 15.0) | 0.47  0.44  0.11 |
| CDR sum of boxes, median (IQR) | 0 (0 - 1.5) | 0 (0 - 2.0) | 0.47 | 15.0 (8.5 - 18.0) | 14.0 (6.4 (17.3) | 0.24 |
| MMSE, median (IQR)^c^ | 27.0 (25.0 - 28.0) | 26.0 (23.8 - 29.0) | 0.98 | 12.5 (0 - 21.0) | 13.5 (2.8 - 18.3) | 0.95 |
| Approved clock drawing test (%)^d^ | 36 (72) | 25 (64) | 0.43 | 3 (9) | 3 (7) | 1.0 |
| NEADL, median (IQR)^e^ | 47.0 (27.5 - 56.5) | 41.0 (19.5 - 57.0) | 0.46 | 5.0 (3.0 - 13.5) | 11.5 (5.0 - 17.0) | 0.10 |
| BADL, median (IQR)^f^ | 19.0 (17.0 - 20.0) | 18.0 (16.0 - 20.0) | 0.16 | 9.0 (4.5 - 14.5) | 11.5 (7.3 - 16.0) | 0.21 |
| Died (%) | 13 (16) | 12 (14) | 0.80 | 33 (41) | 31 (38) | 0.65 |
| SPPB, median (IQR)^g^ | 6.0 (2.5 - 8.0) | 5.0 (2.0 - 9.0) | 0.46 | 2.0 (0 - 3.0) | 1.0 (0 - 4.0) | 0.63 |
| IQCODE, median (IQR)^h^ | 3.06 (3.00 - 3.60) | 3.13 (3.00 - 3.56) | 0.54 | 5.00 (4.88 - 5.00) | 5.00 (4.69 - 5.00) | 0.46 |
| Weight change from index stay in kg, mean (SD)^i^ | - 2.4 (4.7) | - 3.8 (7.1) | 0.28 | - 2.4 (9.0) | - 2.6 (6.8) | 0.95 |
| New nursing home admissions (%) | 6 (11) | 7 (14) | 0.56 | 10 (24) | 11 (24) | 0.96 |
| Incident dementia^j^ | 7 (12) | 3 (6) | 0.34 |  |  |  |
| Re-admissions | 20 (37) | 16 (33) | 0.70 | 6 (16) | 6 (16) | 0.96 |

^a^ To construct the primary endpoint, we normalised CDR and the 10 word test from CERAD into a 0 - 100 scoring (CDR had to be reversed since it is scaled in the opposite direction). CDR weighed 50 % and the immediate and delayed recall parts of the 10 word test weighed 25 % each in the combined measure. No dementia: The primary endpoint was missing in one patient from the acute geriatric and in one patient from the orthopaedic ward. Dementia: The primary endpoint was missing in eight patients from the acute geriatric and in four patients from the orthopaedic ward.

^b^ No dementia: Immediate and delayed recall missing in one patient from the acute geriatric ward and in one patient from the orthopaedic ward. Recognition missing in two patients from the acute geriatric ward and in two patients from the orthopaedic ward. Dementia: immediate and delayed recall missing in eight patients from the acute geriatric ward and in four patients from the orthopaedic ward. Recognition missing in 17 patients from the acute geriatric ward and in 15 patients from the orthopaedic ward.

^c^ No dementia: MMSE was missing in three patients from the acute geriatric ward and in three patients from the orthopaedic ward. Dementia: MMSE was missing in three patients from the acute geriatric ward.

^d^ ≥ 4 points. No dementia: clock drawing test was missing in seven patients from the acute geriatric ward and in ten patients from the orthopaedic ward. Dementia: clock drawing test was missing in seven patients from the acute geriatric ward and in four patients from the orthopaedic ward.

^e^ Dementia: NEADL was missing in two patients from the orthopaedic ward.

^f^ No dementia: Barthel ADL was missing in one patient from the acute geriatric ward. Dementia: Barthel ADL was missing in two patients from the orthopaedic ward.

^g^ No dementia: SPPB was missing in two patients from the orthopaedic geriatric ward. Dementia: SPPB was missing in five patients from the acute geriatric ward and in one patient from the orthopaedic ward.

^h^ No dementia: IQCODE was missing in two patients from the acute geriatric ward and in one patients from the orthopaedic ward. Dementia: IQCODE was missing in one patient from the acute geriatric ward and in three patients from the orthopaedic ward.

^i^ No dementia: Weight missing in 8 patients from the acute geriatric ward and 17 patients from orthopaedic ward. Dementia: Weight missing in 18 patients from the acute geriatric ward and in 28 patients from the orthopaedic ward.

^j^ Based upon consensus in an expert panel (TBW and KE)

SD = Standard Deviation. CERAD = Consortium to Establish a Registry for Alzheimer’s Disease. IQR = Interquartile Range. CDR = The Clinical Dementia Rating scale. MMSE = Mini Mental State Examination. BADL = Barthel Activities of Daily Living. NEADL = Nottingham Extended ADL Index. SPPB = Short Physcial Performance Battery. IQCODE = Informant Questionnaire on Cognitive Decline in the Elderly.
